# Optical fiber tip templating using direct focused ion beam milling

**DOI:** 10.1038/srep15935

**Published:** 2015-11-04

**Authors:** A. Micco, A. Ricciardi, M. Pisco, V. La Ferrara, A. Cusano

**Affiliations:** 1 Optoelectronic Division, Department of Engineering, University of Sannio, I-82100, Benevento, Italy; 2 ENEA, Portici Research Center, P.le E. Fermi 1, 80055 Portici, Napoli, Italy

## Abstract

We report on a method for integrating sub-wavelength resonant structures on top of optical fiber tip. Our fabrication technique is based on direct milling of the glass on the fiber facet by means of focused ion beam. The patterned fiber tip acts as a structured template for successive depositions of any responsive or functional overlay. The proposed method is validated by depositing on the patterned fiber a high refractive index material layer, to obtain a ‘double-layer’ photonic crystal slab supporting guided resonances, appearing as peaks in the reflection spectrum. Morphological and optical characterizations are performed to investigate the effects of the fabrication process. Our results show how undesired effects, intrinsic to the fabrication procedure should be taken into account in order to guarantee a successful development of the device. Moreover, to demonstrate the flexibility of our approach and the possibility to engineering the resonances, a thin layer of gold is also deposited on the fiber tip, giving rise to a hybrid photonic-plasmonic structure with a complementary spectral response and different optical field distribution at the resonant wavelengths. Overall, this work represents a significant step forward the consolidation of Lab-on-Fiber Technology.

The integration of optical fibers with all those materials and structures providing sub-wavelength field confinement and resonant field enhancement, is rapidly expanding the Optical Fiber technology horizons. The addition of novel functionalities to optical fibers requires the integration (in particular on the fiber tip) of photonic and plasmonic crystals, offering remarkable capability of light trapping and manipulation at nanoscale^1^. Fusing together the world of nanotechnologies with optical fibers, is leading to assess a novel and intriguing technology known as ‘Lab on Fiber’, aimed at developing advanced and smart optical probes, especially useful for sensing applications^2^.

Many fabrication processes have been recently proposed to adapt well established nanofabrication strategies to work with unconventional substrates such as the case of optical fiber tip^3^. These approaches can basically be divided into two main categories such as nano-transferring and direct writing. The former consists of preliminary fabrication of nanostructures onto planar substrates, subsequently transferred on the optical fiber tip. The latter relies on direct-write patterning and deposition techniques directly performed on the fiber tip.

In the last years, our group has proposed two different direct-writing approaches (both top-down and bottom-up) for integrating hybrid metallo-dielectric structures on the optical fiber facet. The first one consists of patterning an electron photoresist directly spun on the fiber tip by means of Electron beam lithography^4^,^5^. Successively a thin layer of gold is deposited to obtain metallic nanostructures supporting localized surface plamon resonances. The second approach, on the other hand, is based on self-assembly technique which provides a high production throughput without using high-cost microfabrication facilities^6^. By exploiting the so called breath figures methodology, regular and ordered metallo-dielectric crystals have been integrated onto the optical fiber tip, supporting interferometric effects assisted by surface plasmon excitation at the metallo-dielectric interface.

On this line, in order to enlarge the set of fabrication strategies conferring new functionalities to optical fibers, here we propose a new method essentially based on Focused Ion Beam (FIB) milling, which allows a direct-write controlled nanomachining of the fiber glass.

The potentiality of the FIB instrumentation as an effective tool for micromachining the optical fiber tip has been already demonstrated in the last decade. Preliminary investigations in micro-structuring optical fibre tips with direct focused ion beam milling have been proposed in 2006^7^. In the same year, Iannuzzi *et al.* fabricated a high-sensitivity microcantilever sensor especially useful for atomic force microscopy^8^. By microstructuring the fiber end surfaces, Liberale *et al.* demonstrated a miniaturized fiber based optical tweezer providing a purely optical three-dimensional trap^9^. FIB has been also used to efficiently mill on the end facet of a photonic crystal fiber (PCF) a microchannel enabling the selective fluid filling into a desired pattern of PCF air holes^10^. Moreover, single bowtie nano-aperture patterned at the end of a monomode optical fiber has been demonstrated as new probes for scanning near-field optical microscopy^11^.

Although the FIB milling is especially useful for micro-structuring the optical fiber, so far only a few attempts of direct writing periodic nano-structures onto the fiber tip have been reported. For example, both circular and bow tie-shaped nanoholes arrays were fabricated on gold films deposited on the tips of single mode optical fibers^12^. Similarly, Dhawan *et al.* reported the fabrication of nanopillars and nanoholes in optically thick metallic films. These plasmonic optical fiber probes have been used for chemical sensing and SERS measurements^13^. The above mentioned approaches basically rely on the integration of metallic nanostructures supporting plasmonic resonances, obtained by milling a thin gold layer previously deposited on tips of optical fibers.

Differently from the above mentioned fabrication procedures, here we propose a flexible technique for integrating sub-wavelength resonant structures on the fiber facet, starting from the FIB direct milling of a regular lattice of holes on the optical fiber tip. Once the template is created, any kind of responsive or functional overlays (dielectrics, semiconductors and metals) can be easily integrated through the use of common deposition techniques.

As a particular case of study, here we demonstrate the capability of the proposed approach to realize compact photonic crystal cavities on the fiber tip supporting resonant photonic modes. The fabrication process essentially consists of two main steps: (i) FIB milling is first used to create the desired pattern on the fiber facet; (ii) an optically thick high refractive index layer is successively deposited by means of a Plasma Enhanced Chemical Vapor Deposition (PECVD) process. The resulting structure consists of a ‘double-layer’ photonic crystal slab supporting guided resonances^14^. The excited photonic modes, having most of their energy concentrated in the dielectric layer which is typically lossless in the VIS-NIR wavelength range, have longer propagation lengths than plasmonic modes, thus leading to a large Q factor^15^. The possibility to excite electromagnetic waves strongly confined within the slab and yet capable of coupling to the external radiation, provides an effective and appealing way to sense the external environment. Some studies have demonstrated that sensitivity associated to photonic modes may results higher than that associated to plasmonic modes^16^. Moreover, by exploiting electrical connections combined with semiconductor based components integrated on the fiber tip, it should be possible to develop active filters useful in telecommunication applications^17^.

Although our procedure is here referred with respect to a particular case of study, overall the proposed method is flexible. As a matter of fact, after a suitable optimization of the recipe for patterning the fiber tip, different kind of materials may be deposited to give rise to complex patterned nanostructures without the need to optimize the recipe associated to different materials patterning. Basically, the patterned fiber tip acts as a structured template for the successive deposition of either metallic or dielectric (or a combination of the two) materials able to add new functionalities to our device. With the aim of demonstrating the flexibility of our approach for the development of multilayered-multimaterial nanostructures, as a preliminary proof-of-principle test, we also deposited a thin layer of gold (25 nm thick) on the patterned fiber covered by the nSiO_x_ layer, which is an easy way to engineering the spectral response as well as the field distribution at the resonant wavelengths providing a useful and powerful tool to control light-matter interactions in well defined spatial locations. The deposition of metallic overlays makes possible the excitation of a plasmonic resonance in addition or combination to photonic ones^2^.

This paper is divided as follows: first we introduce the structure under investigation, consisting of a double-layer photonic crystal slab integrated on the fiber tip. With the aid of numerical simulations, we investigate the spectral response and briefly discuss the underlying physics. Successively, we describe the fabrication process together with optical and morphological analysis of the fabricated samples. In order to correctly evaluate the effectiveness of the proposed fabrication procedure, a careful investigation of the undesired fabrication effects on the spectral behavior of the device is presented, with the support of numerical simulations. Before concluding, we finally demonstrated multilayered-multimaterial optical fiber probes achieved thanks to the integration of both dielectric and metallic overlays on the fiber tip.

## Results

### Numerical Design

**Figure 1:**
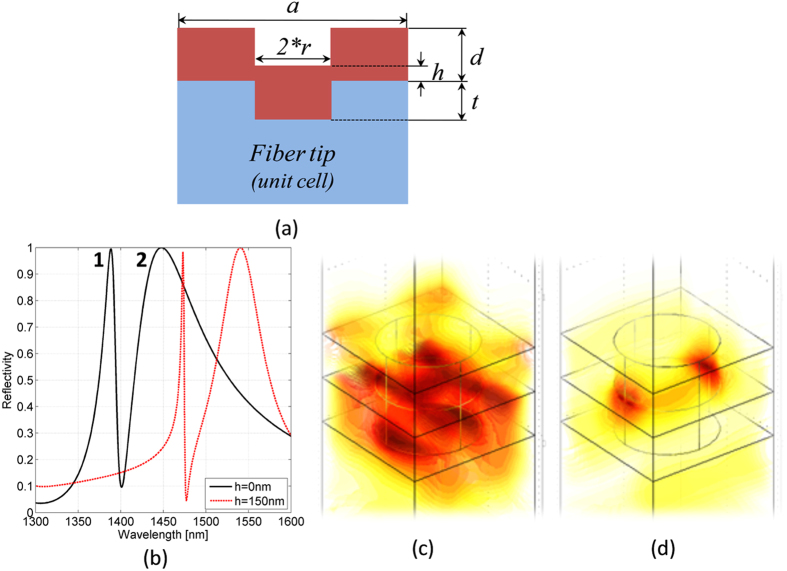
(**a**) Schematic of the double-PC slab structure integrated on the fiber tip; (**b**) theoretical reflectance spectrum pertaining to device of Fig. 1(a) achieved with the following parameters *a* = 900 nm, *r*/*a* = 0.35, *t* = *d* = 300 nm, with *h* = 0 nm (solid black line) and *h* = 150 nm (red dashed line); (**c**,**d**) electric field intensity maps relative to resonances 1 and 2 calculated at wavelengths corresponding to reflectance peaks (at 1388 and 1448 nm).

The structure under investigation, schematically shown Fig. 1(a), is a ‘double-layer’ photonic crystal structure; basically the bottom slab consists of a patterned glass layer (square lattice of holes with pitch *a* and radius *r* and depth *t*), whose holes are filled with nSiO_x_; the top slab is a nSiO_x_ layer with thickness *d* patterned with air holes.

When such a structure is illuminated in out-of-plane configuration, as the case of single mode fiber illumination in the paraxial propagation regime, photonic resonances are excited due to the coupling between the scattered waves and the modes supported by the PC slabs. In order to numerically analyze this structure, we performed some simulations by using the commercial software COMSOL Multiphysics (RF module) based on the finite element method^18^. Details on the numerical simulations can be found in the Supplementary Information. The refractive index of fiber fused silica is set to 1.45 while refractive index of nSiO_x_ has been modeled according to spectroscopic ellipsometry measurements performed on films deposited on glass (2.144@1450 nm and 2.135@1650 nm).

In Fig. 1(b) we plot the theoretical reflectance spectrum of fiber tip device of Fig. 1(a) for *t* = 300 nm and *d* = 300 nm (solid black line); the pitch (*a* = 900 nm) and the holes radius (*r* = 315 nm, corresponding to a filling factor *r*/*a* = 0.35) have been chosen in order to set the resonances in a wavelength range of single mode operation of standard optical fiber.

Two resonances arise at 1388 nm (resonance 1) and 1448 nm (resonance 2). The electric field distributions at the resonant wavelength relative to resonance 1 and 2 are plotted in Fig. 1(c,d) respectively. Resonance 1 (at lower wavelengths) has most of its electric field concentrated in the bottom slab, while resonance 2 (at higher wavelengths) has an electric field mostly localized at the interface between the two slabs.

With the aim of evaluating the effects of the dielectric layer conformal (isotropic) deposition on the device performances, we also analyze the case where a uniform nSiO_x_ layer of thickness *h* = *d*/2 = 150 nm is interposed between the two PC slabs. The reflectance spectrum is shown as solid red line in Fig. 1(b). In this case, a red shift of 85 and 92 nm for resonances 1 and 2 respectively occurs. Moreover, consistently with the guided resonance theory, the resonance red-shift is combined with a Q-factor enhancing, due to the effective refractive index increasing of the guiding layer, which guarantees a longer resonance lifetime^14^. Specifically Q-factors (defined as the ratio between resonance peak wavelength and full width half maximum FWHM) move from 73 to 268 and from 13 to 23 for resonance 1 and 2 respectively.

It is worth underlining that, inherently to the pursued approach, the numerical simulations clearly refer to an infinite pattern since periodic boundary conditions are used.

### Fabrication and Morphological Analysis

According to the design parameters, and in order to evaluate the influence of the finite size of the pattern, we have fabricated two samples with *d* = *t* = 300 nm and patterned areas of 14.4 × 14.4 μm^2^ (small area) and 28.8 × 28.8 μm^2^ (large area - maximum exposable), corresponding to replica of 16 and 32 unit cells, respectively. The fabrication process is described in the methods section.

**Figure 2:**
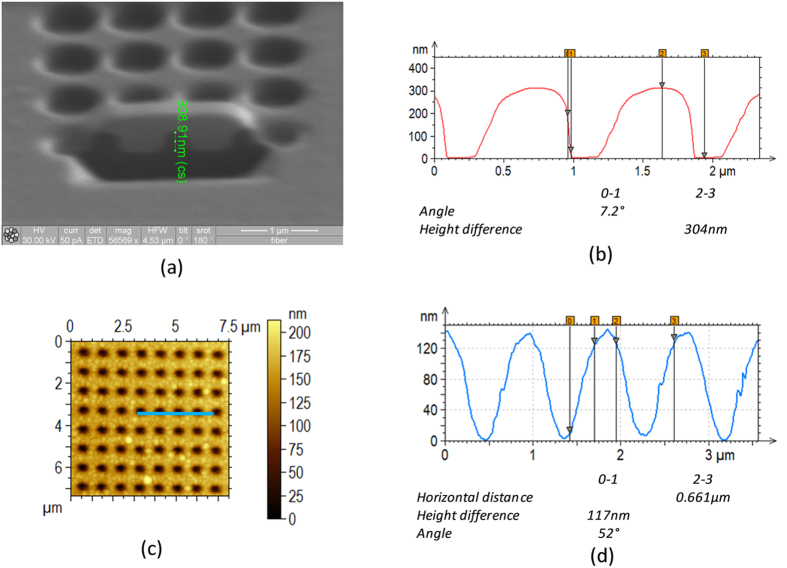
(**a**) Scanning ion microscopy image of a square lattice of holes milled on the optical fiber tip used for recipe optimization; (**b**) AFM profile of (**a**); (**c**) AFM top view image of holes after nSiO_x_ overlay deposition, zoomed in for better view; (**d**) AFM profile of the scan line indicated in (**c**).

In order to investigate the shape of nanoholes, cross sections are performed on the optical fiber tip always using FIB. Before cross-sectioning, a platinum layer is deposited on the features both covering milled holes and protecting surface from ion bombardment. A scanning ion microscopy image of a cross section is shown in Fig. 2(a), where it is evident the hole shape and its estimate depth. The FIB milling recipe has been optimized for achieving the desired hole depths and vertical sidewalls. In order to further verify the compliance to the design specifications, AFM measurements (by means of an atomic force microscope Agilent 5420) have been carried out on the optical fiber tip, and the results are shown in Fig. 2(b). Whenever AFM scans can effectively measure the holes width (i.e. 635 ± 5 nm) and depth (i.e. 304 ± 3 nm), the sidewall profile evaluation is intrinsically affected by AFM tip-convolution artifact due to the shape of the tip, resulting in asymmetry in the hole profile and an overestimate of conical angle in the less steep sidewall^19^. By considering both AFM and FIB images, the hole conical angle can be realistically estimated to be less than 10 degrees (about 7.2 ± 1.3°).

After the FIB milling, a sub micrometer thick (~300 nm) nSiO_x_ is directly deposited on the fiber tip by means of PECVD procedure (see Methods). As before, morphological characterization has been carried out directly on the fiber tip again. Figure 2(c,d) show both top-view and the cross profile of the device after the overlay deposition. The conic angle is estimated to be around 50 degrees (52 ± 3.5°), significantly larger than that relative to the bottom slab. Moreover, the hole depth is found to be 167 ± 7 nm for a deposited nSiO_x_ thickness of 300 nm, meaning that the conformal overlay deposition tends to ‘close’ the patterned holes thus reducing depth and increasing conic angle with respect to the bottom slab.

### Optical characterization

We have conducted spectral reflectance measurements in order to characterize the resonant behavior of the fabricated device. Details on the characterization procedure are described in Methods section.

**Figure 3:**
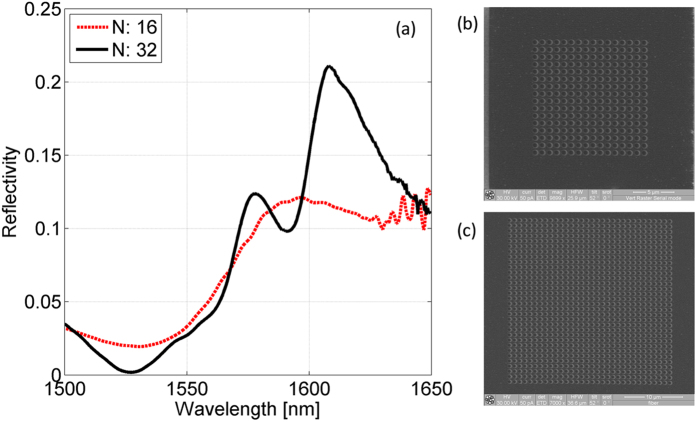
(**a**) Experimental reflection spectra obtained with patterned areas of 14.4 × 14.4 μm^2^ (red dotted line) and 28.8 × 28.8 μm^2^ (black solid line). The SEM images (top view) of the characterized probes with small and large patterned areas are plotted in (**b**,**c**) respectively.

Figure 3 shows the measured reflection spectra pertaining to the samples with small (red dotted line) and large (black solid line) patterned area. The SEM images (top view) of the characterized samples are also shown in Figure 3(b,c). As it can be observed, when the patterned area is 14.4 × 14.4 μm^2^, the number of periods is not sufficient to give rise to a significant resonant phenomenon and no noticeable spectral features appear in the spectrum. When the patterned area is increased up to 28.8 × 28.8 μm^2^, two resonances located at 1578 nm and 1608 nm arise according to our numerical predictions with Q-factor of 66 and 30 respectively. However, even in this second case, the discrepancies in terms of reflectivity peak intensity and Q-factor between numerical and experimental results still remain, due to the finite size of the patterned area. As a matter of fact, both the visibility and Q-factor of guided resonance modes vary with the size of the photonic crystal; this variation is due to losses caused by scattering of in-plane propagating modes at lattice boundaries and eventual coupling of incident light to fully guided modes that exist in the homogeneous slab outside the lattice boundaries^20^.

In addition to a resonance visibility reduction, the experimental resonances shown in Fig. 3 are significantly red-shifted (~200 nm) with respect to the numerical counterparts. With the aim of explaining the reasons for that variation, we carried out some simulations in which we tried to take into account the real morphology of the fabricated samples. Three different ‘defects’, intrinsic to the proposed fabrication procedure, have been taken into account: (i) the conical shape of the patterned holes, (ii) the gallium ion doping of the silica after the milling process and (iii) the shape (in terms of both conic angle and depth) of the top slab holes, which is mostly influenced by the deposition step.

### Numerical fitting

In the following we analyze how the spectral response of our device is affected by the above mentioned fabrication defects, first individually and then combined together in order to achieve a numerical fitting of the experimental results.

**Figure 4:**
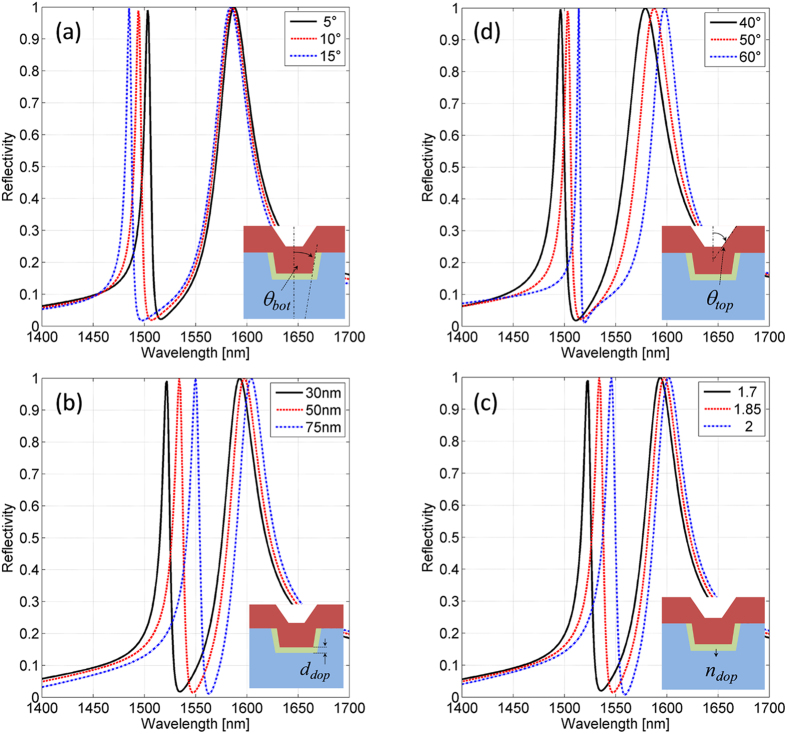
Theoretical reflection spectra as a function of: (**a**) *θ_bot_* with fixed *θtop* = 50° (no ion doping effect); (**b**) *d_dop_* with fixed *θ_top_ *= 50° *θ_bot_* = 5°, *n_dop_* = 1.85; (**c**) n_dop_ with fixed *θtop* = 50°, *θ_bot_* = 5°, *ddop* = 50 nm; (**d**) *θ_top_* with fixed *θ_bot_* = 5° (no ion doping effect).

Specifically, as regards point (i) and according to the morphological analysis and resumed in Fig. 2(a), the conical angle *θ*_*bot*_ of the hole sidewalls is made to vary in the range from 5 to 15 degrees. Concerning (ii), it is known that the silica doping during the FIB milling procedure results in an increasing of the refractive index (RI); the amount and the depth of the RI change depends on the ion energy^21^. Change of RI imaginary part have not been evaluated. In order to estimate this effect, we include in our model a region underneath the patterned hole (see the inset of Fig. 4(b)) where the silica RI of the fiber *n*_*dop*_ is increased up to values in the range 1.7–2 for a depth *d*_*dop*_ comprised in the range from 30 to 75 nm^21^. Finally, with regard to (iii) and according to the morphological measurements shown in Fig. 2(b,c), in our calculations the top slab hole depth (*d*_*top*_) is kept fixed to 170 nm, while small perturbations (10 degrees) of *θ*_*top*_ around 50 deg have been considered.

The computed reflection spectra as a function of the above mentioned parameters are shown in Fig. 4 (details in the figure caption). As it is possible to notice, resonance at smaller wavelength (resonance 1) is mostly influenced by both ion doping in the silica region and not perfectly perpendicular side-walls of the milled holes. Resonance at longer wavelength (resonance 2), instead, is mostly affected by the shape of the nSiO_x_ overlay. Therefore, as expected, we found that the fabrication defects have a different influence on the two resonances according on their electromagnetic field distributions (cfr Fig. 1(c,d)). If the maximum of the electric field intensity is concentrated around the areas influenced by the fabrication defect, then a substantial shift of the related resonant wavelength occurs.

In particular, as it is possible to notice in Fig. 4(a), increasing the value of *θ*_*bot*_ causes a blue shift of resonances 1 due to a decreasing of the effective refractive index of the guided mode excited in the bottom slab. Basically the larger *θ*_*top*_ the smaller the quantity of high refractive index (i.e. nSio_x_) filling the hole slab, since the filling factor decrease as we move inside the fiber. A similar consideration applies for spectral behavior as a function of *θ*_*top*_ plotted in Fig. 4(d). In this case, however, an opposite trend is observed; both the resonances exhibit a red shift (although with different sensitivities) in line with an increasing of the effective refractive index of the top slab. Furthermore, as already mentioned before, in both Fig. 4(a,d), the resonance blue-shift/ red-shift is always combined with a Q-factor decreasing/increasing respectively. In fact, when the effective refractive index increases, then the resonance mode is ‘guided’ in a higher effective refractive index layer, with a consequent red shift (and a Q-factor enhancement). A detailed quantitative analysis goes beyond the scope of this work.

Overall, the defect affecting more strongly the spectral response is the ion doping. In fact, as the ion doping expands (spreads) inside the optical fiber, resonance 1 (having an e.m. field distribution localized in the bottom slab) undergoes to a significant red shift, pushing the resonant wavelength close to that of resonance 2 that is by its nature less sensitive to modifications occurring inside the fiber due to the FIB milling. Clearly, the ion doping may also affect the imaginary part of the silica, thus increasing the optical losses.

**Figure 5:**
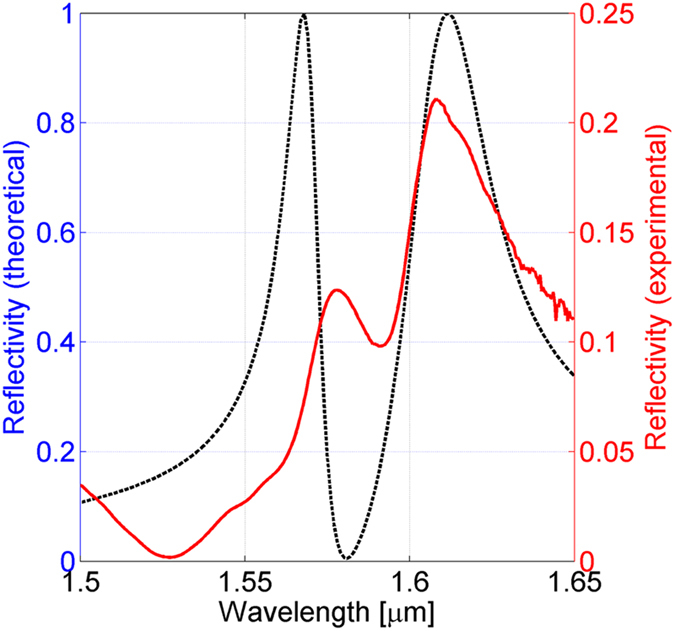
Comparison between experimental and theoretical (numerical fitting) reflection spectra of the fabricated device.

Finally, except for further fabrication tolerances, by considering all the effects discussed so far and putting them together in the same model with *θ*_*top*_ = 50°, *θ*_*bot*_ = 5°, *d*_*dop*_ = 75 nm, *n*_*dop*_ = 2, a fairly good agreement between experimental and numerical results is achieved as shown in Fig. 5.

The experimental Q-factors are 66 and 30 for resonance 1 and 2 respectively. FWHM of resonance 1 has been calculated by considering twice the half FWHM evaluated on the rising edge. From the numerical model simulations Q-factor of 108 and 47 are obtained. The discrepancy (39% and 36% for resonance 1and 2 respectively) with respect to the numerical prediction are principally due to the finite extent of the pattern on the fiber tip. In fact these results are consistent with those found in refs. 20 and 27. Other reasons of such a disagreement can be due to fabrication tolerances such as the hole disuniformity and boundary roughness.

### Towards multilayered-multimaterial optical fiber probes

Before concluding, in this section we provide a preliminary proof-of-principle test about the flexibility of the proposed fabrication approach for achieving multilayered-multimaterial enabling the tailoring of the spectral response of the final device, as well as the control of the field distribution at the resonant wavelengths. As already mentioned in the introduction, in addition to a suitable choice of the lattice geometrical parameters, resonance engineering can be also achieved thanks to the integration, onto the previously patterned optical fiber tip, of different materials with specific optical properties^2^.

The integration of metals leads to the creation of metallic nanostructures on the optical fiber tip supporting both propagating and localized surface plasmon resonances (SPR and LSPRs) which exhibit high local electromagnetic field intensities confined at sub-wavelength scale. Plasmonic modes are principally exploited for monitoring chemical variations (such as molecular binding events) occurring on the metal surface. Different Lab on Fiber based label free biosensors have been reported so far^22^,^23^,^24^. Moreover, LSPRs excited on the fiber tip could be exploited also for surface enhanced Raman spectroscopy (SERS) which involves the study of molecules adsorbed to the sensor surface^12^,^25^,^26^.

Here we analyze a structure where a double layer deposition (of both dielectric and metallic overlays) is made, generating a multilayered-multimaterial optical fiber probe. This structure can in principle support both photonic and plasmonic (or hybrid) resonances, with different electric field distributions allowing the control of light-matter interactions in well defined spatial depending on the specific application.

**Figure 6:**
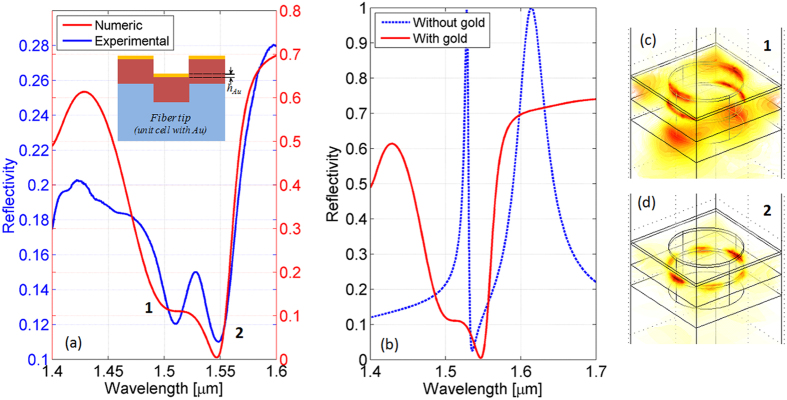
(**a**) Comparison between experimental (solid blue line) and numerical (solid red line) reflection spectra pertaining to device schematized in the inset, achieved with the following parameters *a* = 900 nm, *r*/*a* = 0.35, *t* = 300, *d* = 200 nm, *θbot* = 5°, *θ_top_* = 50°. The gold overlay has a thickness of 25 nm. (**b**) Comparison between numerical reflection spectra with (solid red line) and without (blue dotted line) the gold overlay. Electric field intensity maps evaluated at reflectance dips wavelengths (1510 (**c**) and 1548 nm (**d**)).

Specifically, as a first case of study, we have fabricated another sample with the following parameters *a* = 900 nm, *r/a* = 0.35, *t* = 300, *d* = 200 nm (patterned area 28.8 × 28.8 μm^2^), by using the same fabrication process discussed above. Successively, a 25 nm thick gold overlay was deposited on the top of nSiO_x_, giving rise to the structure schematized in the inset of Fig. 6. The measured reflectance spectrum is shown in Fig. 6(a) as solid blue line. In the same figure we also show as solid red line the numerical counterpart achieved by using the model taking into account the fabrication defects, with the following parameters, *a* = 900 nm, *r/a* = 0.35, *t* = 300, *d* = 200 nm, *θ*_*bot*_ =5°,  *θ*_*top*_ = 50°. For completeness, we also show in Fig. 6(b) a comparison between numerical reflection spectra before and after the gold overlay deposition. First of all we notice that, differently from the previous case, the presence of the gold layer has the main effect of increasing the reflection baseline, with the consequent ‘inversion’ of the spectral response. With reference to the experimental spectrum, the relatively high reflectivity baseline is interrupted by two resonant dips at 1510 and 1548 nm, in fairly well agreement with the numerical predictions. It is also important to underline that each change in the resonance wavelength and visibility is accompanied with modifications of the electric field distribution inside the nanostructure (see Fig. 6(c,d)). In this particular case, resonance at smaller wavelength is characterized by having a strong electric field localization around the top gold slab, representing a good solution for label-free chemical sensing purposes.

Although no design and optimization were involved, the proposed sample represents a convincing demonstration of the potentialities offered by the proposed fabrication procedure. Enlarging the set of materials integrated on the fiber tip can further improve the light flow control and confinement, conferring to the final device novel features in terms of functionalities and performances.

Furthermore, the just discussed case allows to quantitatively estimate the scattering losses, because the presence of the uniform gold layer on the top of optical fiber enhances the back reflected light intensity and minimizes the transmission. By observing the measured reflection spectrum shown in Fig. 6, we found that the reflection baseline in the investigated wavelength range, away from the resonant wavelengths, does not exceed the 30% (instead of 70% in the numerical case) meaning that most of the light is lost in the in-plane direction and is not coupled back in the out-of-plane direction (i.e. reflection).

## Discussion

In conclusion, we have proposed a new fabrication path for realizing Lab-on-Fiber devices through a direct writing approach. The method is based on focused ion beam milling directly performed on the optical fiber tip followed by an overlay deposition. The proposed technique is flexible since after milling the fiber tip both dielectric and metallic materials could be deposited, and eventually combined together to give rise to complex metallo-dielectric structures, with different spectral response.

For evaluating the validity of the proposed fabrication procedure, in this work, we have integrated on the fiber tip a “double layer” dielectric photonic crystal slab supporting guided resonances, achieved by depositing a high refractive index (nSiO_x_) material layer on the optical fiber tip previously milled by FIB. The resulting device has been characterized from both optical and morphological point of view. Our experiments demonstrate the effectiveness of the proposed fabrication method (the results of a fabrication trial aimed at assessing repeatability and success rate of the fabrication method are included in the supporting information).

Furthermore, in order to demonstrate the capability of our approach to conveniently create multilayered-multimaterial fiber optic nanoprobes, a metallo-dielectric nanostructure has been successfully realized on the fiber tip. This structure supports both photonic and plasmonic (or hybrid) resonances, enabling the spectral response engineering as well as the control of the field distribution at the resonant wavelengths, providing a useful and powerful tool to control light-matter interactions in well defined spatial locations depending on specific applications. The choice of the functional material integrated on the structured fiber tip clearly depends on the specific application. For example metallic nanostructures integrated on the optical fiber tip and supporting LSPRs could be exploited for chemical and biochemical sensing field, giving rise to advanced optical fiber probes able to potentially perform point-of-care diagnosis in remote locations such as the human body, for *in vivo* applications. Semiconductors, on the other way, are preferably used for developing tunable devices, by exploiting the strong electro-optic effect.

Overall, our results demonstrate that the FIB milling is a rapid, flexible and versatile tool to nanostructure the optical fiber tip. We have studied the influence of the FIB process on the final device characteristics. In particular we have found that both angled sidewalls of patterned holes and above all the gallium ions implantation into the fiber glass (that causes a significant increasing of the silica refractive index) influence the spectral response of the device. Depending on the electromagnetic field distributions, the excited resonances experience different spectral changes. All these effects have to be carefully taken into account during the design phase in order to guarantee a successful development of the device. However, there are still several challenges to address for the successful implementation of this technique in advanced lab on fiber devices with well defined spectral features (in terms of visibility and Q-factor). In addition to all the fabrication defects, intrinsic to the FIB process (such as the ion doping and the angled sidewalls of the patterned holes), that it is possible to consider and model in the design phase, the major obstacle is represented by the limited size of the patterned area. This aspect plays a crucial role on the spectral characteristics of the fabricated device, significantly limiting the probability of achieving high Q resonances. In fact, the patterned area size is directly related to the in-plane extension of the excited guided modes, that, if not suitably confined, they can couple to fully guided modes propagating in the homogeneous slab outside the lattice boundary, thus causing scattering losses. Due to the propagative nature of the guided resonances, this issue is particularly critical when the refractive index contrast of the integrated photonic crystal slab is low, or in other words, when low refractive index materials are deposited on the patterned fiber tip.

To mitigate these drawbacks, one approach that could be used is to perform write-field alignment procedure, in order to definitely extend the patterned area in such a way to contain a sufficient number of periods, ensuring good resonance visibility high Q-factor. Particular care must be taken in this case to the stitching errors that can cause the abrupt interruption of the pattern periodicity.

Another possible strategy would be to utilize 1D Distributed Bragg Reflectors (DBRs) as already reported in references^20^,^27^. DBRs, designed with the bandgap at the resonant mode wavelength, are able to locally confine the excited guided resonances, thus limiting the scattering losses associated to propagation outside the structured areas.

Although the above mentioned fabrication tricks result in greater device yield and improved device quality, the realization of these complex pattern on the fiber tip is more time consuming, due to the serial nature of the FIB-based fabrication paths.

Finally, the proposed fabrication approach represents a simple and effective route to create active fiber tip adding new functionalities to optical fibers and enriching the portfolio of fabrication strategies necessary to empower the potential of the Lab on Fiber vision.

## Methods

### Fabrication

A standard single mode fiber (Corning SMF-28) is cleaved to obtain a smooth surface. A segment (of about 7 cm) of the fiber is rinsed with ethanol and placed on a proper sample holder. Electron beam evaporation is used to coat the fiber tips with 50 nm of gold to avoid electrostatic charging during FIB milling. A Focused Ion Beam (FIB) milling (Quanta 200 3D FEI instrument) is used to fabricate the square lattice of holes in the glass. Before patterning the holes matrix, the gold layer is removed onto an area of about 40 × 40 μm^2^, in correspondence of the fiber core. Beam currents of 50 pA and accelerating voltages of 30 kV energy are chosen. The desired pattern is milled by rastering the ion beam and employing a source text code where all the circular holes are defined in terms of diameter and lattice coordinates. The magnification is varied between 3500 and 7000 depending on the desired minimum feature size.

After the FIB milling, the fiber is placed inside a VHF-PECVD chamber using a suitable holder which locates the fibers in the correct position. A sub micrometer thick (~300 nm) nSiO_x_ is deposited by using an ultra-high vacuum cluster tool deposition system (MVSystems Inc). The distance of the fiber tips from the electrode is less than 15 mm. The deposition takes place at a temperature of 150 °C, pressure of 200 mTorr, power of 5 W, using pure silane, with a deposition rate of about 4 Å/s.

### Optical Characterization Setup

Spectral measurements of the fiber sample are carried out by using a broadband optical source and redirecting the reflected light (via a 2 × 1 directional coupler) to an optical spectrum analyzer (Ando AQ6317C). To retrieve the sample reflectance, the measured spectrum is then normalized with respect to that pertaining to a fiber optic reference mirror, fabricated by depositing, on the fiber tip, a 150 nm-thick gold film.

## Additional Information

**How to cite this article**: Micco, A. *et al.* Optical fiber tip templating using direct focused ion beam milling. *Sci. Rep.* **5**, 15935; doi: 10.1038/srep15935 (2015).

## Supplementary Material

Supplementary Information
