# Supplementary material for: Optical fiber tip templating using direct focused ion beam milling
Source: Sci Rep. 2015 Nov 4;5:15935. doi: 10.1038/srep15935 (PMC4632123; doi:10.1038/srep15935)
Supplement: Supplementary Information [file srep15935-s1.pdf]

Supplementary information

Optical fiber tip templating using direct focused ion beam milling

A. Micco[1], A.Ricciardi[1], M. Pisco[1], V. La Ferrara[2], A. Cusano[1,*]

[1]Optoelectronic Division, Department of Engineering, University of Sannio, I-82100, Benevento, Italy
[2]ENEA, Portici Research Center, P.le E. Fermi 1, 80055 Portici, Napoli, Italy

*Correspondence

Tel: +39 0824 305835, E-mail: acusano@unisannio.it

1. Numerical Methods

The simulations were carried out using the finite-element method (FEM) with the commercial modeling tool COMSOL Multiphysics®- RF Module (COMSOL Inc., Burlington MA, USA).

To numerically retrieve the reflectance of the photonic crystals using FEM, we restricted the computational domain to one quarter of cell. The quarter of cell was transversely terminated with two horizontal, perfectly electric-conducting and two vertical, perfectly magnetic-conducting walls to simulate a normally incident plane-wave with a vertically polarized electric field. On the bottom of the slab, we assumed a homogenous glass substrate (the optical fiber), while on the top, we used air (the surrounding medium).

The double layer photonic crystal structure on the fiber top is drawn according to the morphological analysis results by taking into account the fabrication defects. The double layer structure, schematically depicted in the figure 1(a) of the main text, is modified in order to take into account the conical angle of the holes sidewalls. Additionally the ion doping is taken into account by including a thin region underneath the patterned hole with a higher refractive index than the glass one, as schematically represented in figure S1.

The values of the physical and geometrical parameters used in the numerical simulations have been promptly indicated in the main text for each reported numerical result.

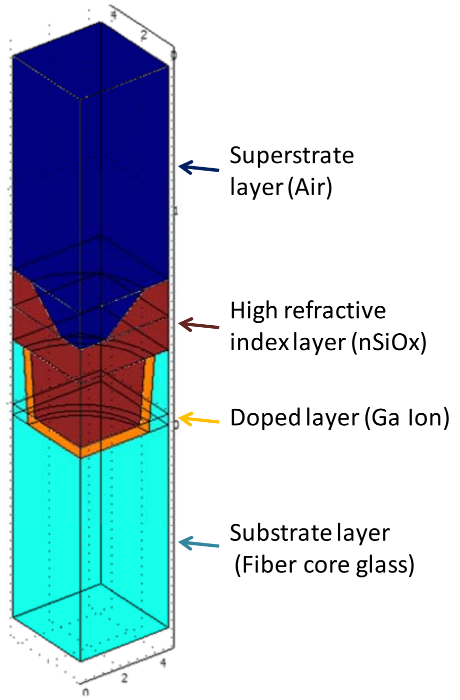

Figure S1. Computational domain for the numerical simulations.

1.1. Influence of the hole depht in the design

The proposed optical platform, constituted of a double layer photonic crystal structure on the optical fiber tip, offers intrinsically high degrees of freedom for its design.

By way of example, the depth of the patterned holes can be efficiently used to finely tune the spectral position of the resonances. In figure S2, we report the results of few numerical simulations aimed to illustrate the effects of the array holes depth on the samples reflectance. For the numerical simulations, we used the overlay thickness d=300 nm; the pitch a=900 nm and the holes radius r=315 nm. As evident in figure S2, both resonances slightly red-shift for increasing holes depth (ranging from 200nm to 300nm), without meaningful changes in the Q-factors (73 and 13 for the left and right resonances respectively). The sensitivity of the resonance spectral position with respect to the holes depth varies in virtue of the guided resonances features. Being the electric field of the resonance 1 mostly concentered in the bottom slab (see the main text), it results more sensitive to the holes depth.

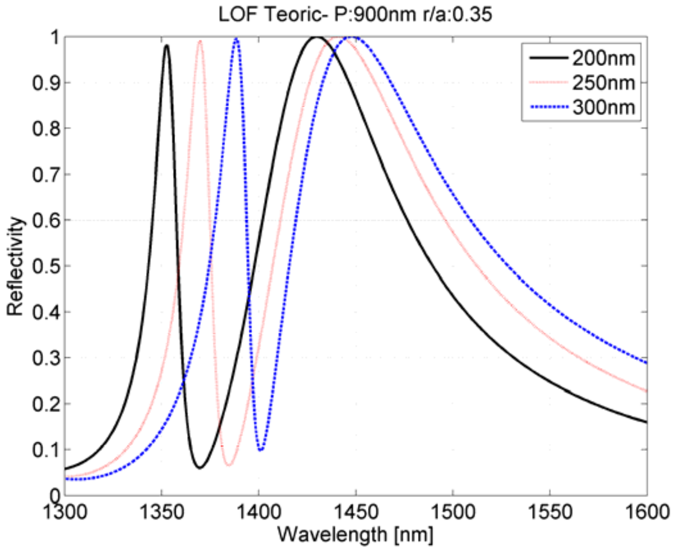

Figure S2. Theoretical reflection spectra with overlay thickness d=300 nm; pitch a=900 nm, holes radius r=315 nm as a function of holes depth t (t=200nm, 250nm and 300nm)

2. Optical Measurements

The spectral reflectance measurements were carried out by illuminating the fiber tip with a broadband optical source (obtained by combining four different SLED operating in the NIR and redirecting the reflected light (via a 2 x 1 directional coupler) to an optical spectrum analyzer (Ando AQ6317C). In addition, to compensate for intensity variations of the source vs wavelength, the sample reflectance was normalized using a fiber-optic reference mirror fabricated by depositing a 150 nm-thick gold film on the tip of a standard single-mode fiber. No polarization control has been

used in our experimental setup. The schematic of the characterization setup is reported in the Figure S3.

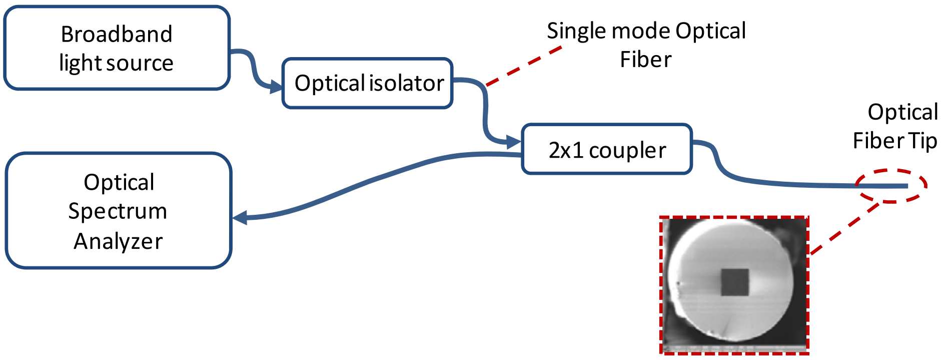

Figure S3. Schematic of the experimental setup used for the reflectance spectra characterization.

3. Repeatability of the fabrication method

In order to assess the repeatability and success rate of the proposed fabrication method, we tried to integrate several subwavelength resonant structures on the fiber tip by exploiting the fabrication process described in the method section. As test bench we used the aforementioned 'double-layer' photonic crystal slab on the optical fiber tip supporting guided resonances.

Specifically, by FIB milling we obtained a regular holes pattern composed of 20x20 holes in square array spaced 850 nm apart. The holes are featured by a diameter of 425nm and a depth of 200nm.

In Figure S4 we show the scanning electron microscope (SEM) top view images of five samples fabricated according to the same recipe. In particular, in figure S4a we show a large view of the fiber tip. The light area is the one covered by gold, whereas the dark area is the realized pattern. In Figures 3b and 3c, we show a magnified SEM image and an atomic force microscope (AFM) image of the structure, respectively, allowing for accurate study of the topography of the patterned region. So, in the following figure S4-b and in the figuresS4-c,S4-d,S4-e andS4-f, we show a magnified SEM image of the same structure and of other four samples, respectively, allowing for accurate study of the topography of the patterned region.

The reported results demonstrate the ability of the proposed method to synthesize regular and ordered templates on the tips of standard, single-mode optical fibers. After the FIB milling a nSiOx layer as thick as 300nm is deposited on the fiber tip by PECVD, by covering the holes pattern in a conformal fashion.

All the fabricated samples have been then characterized spectrally and the experimental results are shown in figure S5 for comparison together with the numerical prediction reflectance spectrum (green dotted line). The numerical spectrum has been obtained by considering the model discussed

in section x of the supporting information, by taking into account the fabrication defects (i.e. angled sidewalls and ion doping) intrinsic to our fabrication process.

The success rate of the fabrication method is 100% since no fault has been recorded. Even e if the reflectances are systematically featured by the fabrication defects discussed and analyzed in the main text, a good repeatability of the fabrication process can be clearly appreciated.

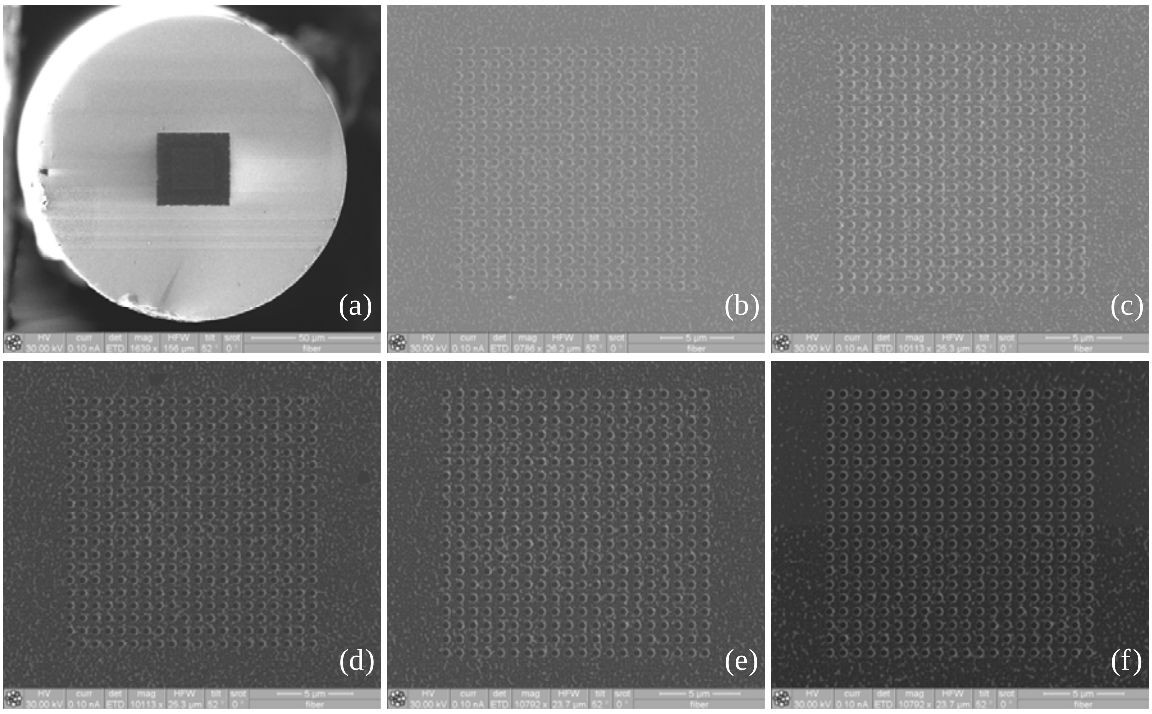

Figure S4. Scanning electron microscope (SEM) top view images of five samples

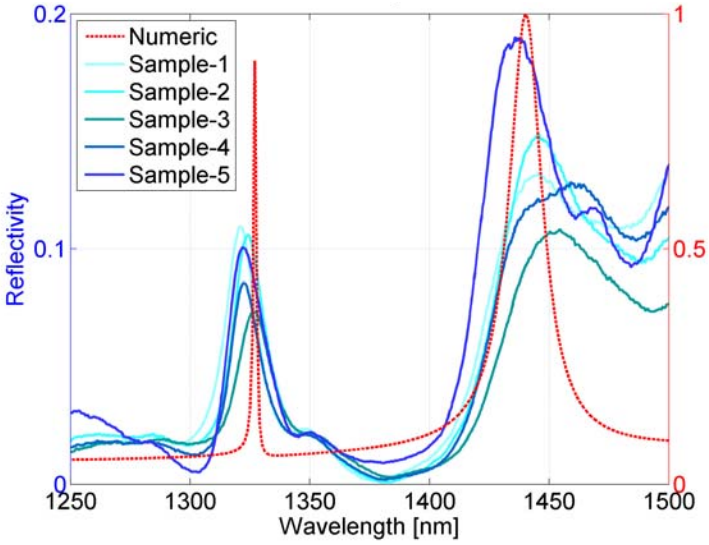

Figure S5. Reflection spectra of the five fabricated samples compared with the numerical counterpart (red dotted line)
